# Supplementary material for: Influenza virus infection expands the breadth of antibody responses through IL-4 signalling in B cells
Source: Nat Commun. 2021 Jun 18;12:3789. doi: 10.1038/s41467-021-24090-z (PMC8213721; doi:10.1038/s41467-021-24090-z)
Supplement: Supplementary file 1 — Supplementary Information [file 41467_2021_24090_MOESM1_ESM.pdf]

Supplementary Fig. 1

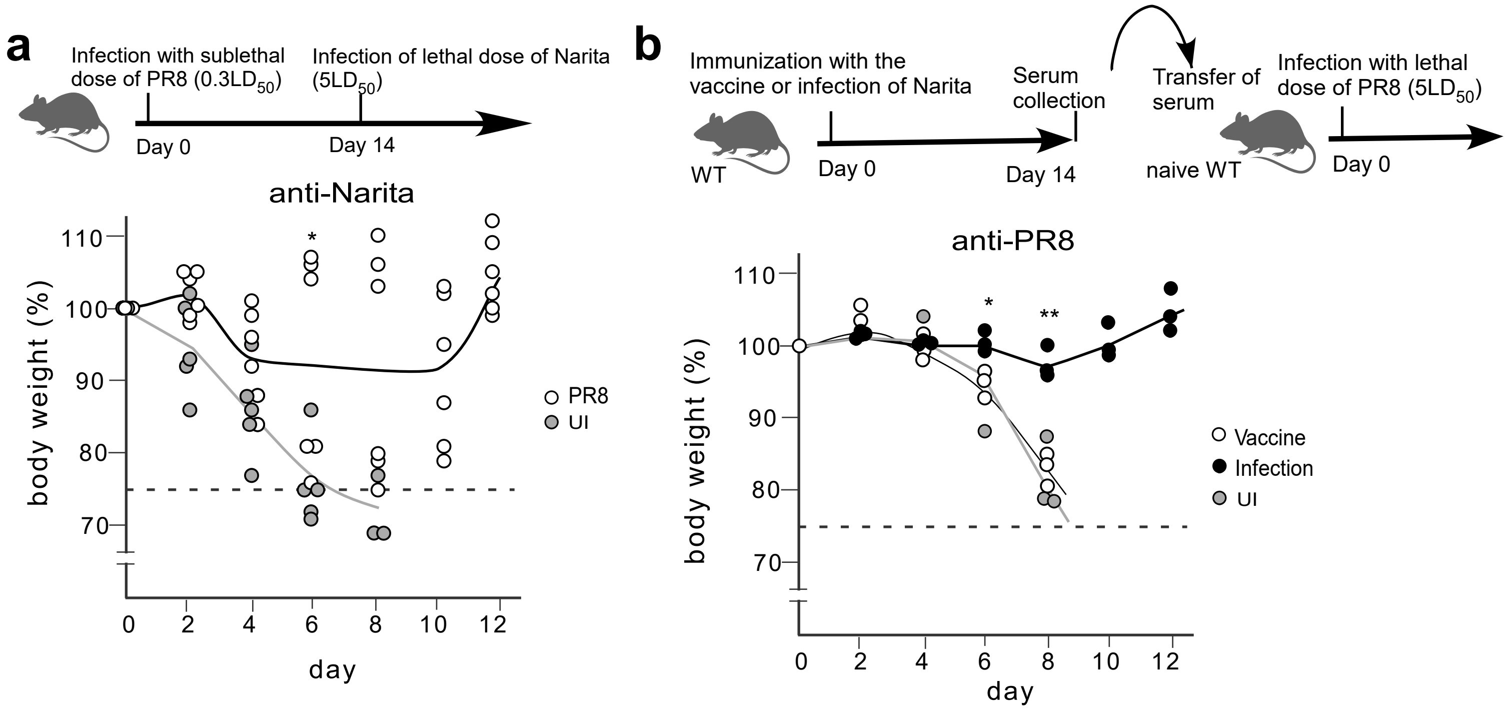

Supplementary Fig. 1 Protection against heterotypic virus infection

a, Unimmunized (UI) mice (gray dots, n=4) and mice immunized by intranasal infection (white dots) with A/PR/8/1934 (n=6) were challenged intranasally with a lethal dose of A/Narita/1/2009. The body weight change of mice was monitored until 12 days post-infection. **b**, Mice immunized by the inactivated vaccine (vaccine n=3 (white dots)) or the intranasal infection (infection n=3 (black dots)) of A/Narita/1/2009 were challenged intranasally with a lethal dose of A/PR/8/1934. The body weight change of mice was monitored until 12 days post-infection. Missing symbols mean that the mice were euthanized, as described in Fig.1. *$p<0.05$, **$p<0.01$ by unpaired, two-tailed *t*-test (UI group with PR8 group in **a**, Vaccine group with Infection group in **b**).

Supplementary Fig. 2

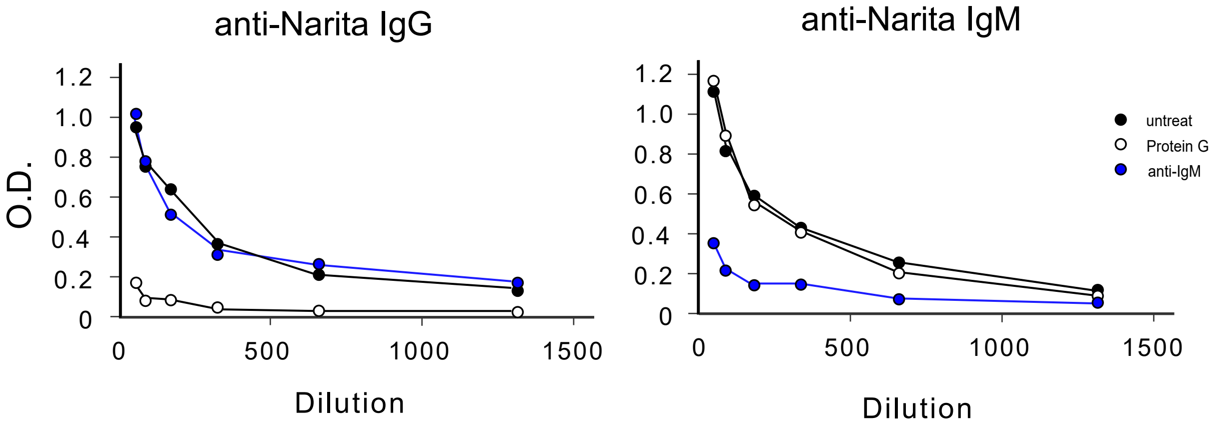

Supplementary Fig. 2 Depletion of IgG or IgM antibodies from BALF
BALF was passed through Protein-G (white) or anti-mouse IgM antibody (blue) conjugated Sepharose columns. Antibodies specific for Narita-HA were measured by ELISA. Data indicates OD values.

Supplementary Fig. 3

Single

Clone	*Igh*	*Igk*
**SP66**	V1-15 D2-3 J3	V4-59 J5
**SP102**	V1-15 D2-3 J3	V4-59 J5
**SP132**	V1-15 D2-3 J3	V4-59 J5
SP143	V1-26 D4-1 J2	V8-30 J5
SP53	V1-26 D2-1 J3	V4-79 J5
SP72	V1-34 D1-1 J3	V4-53 J5
SP67	V1-36 D1-1 J1	V14-111 J2
SP87	V1-36 D4-1 J4	V6-17 J1
SP77	V1-50 D4-1 J2	V14-111 J5
SP103	V1-50 D1-1 J1	V4-72 J5
**SP128**	V1-64 D2-4 J4	V8-30 J2
SP110	V1-69 D1-1 J1	V10-94 J5
**SP120**	V1-72 D4-1 J1	V9-120 J1
SP9	V1-74 D2-3 J3	V11-125 J5
SP126	V1-81 D1-1 J4	V4-57-1 J4

LAH

Clone	*Igh*	*Igk*
**SP109**	V14-1 D2-4 J3	V4-59 J2
**SP36**	V14-2 D2-3 J1	V14-100 J5
**SP111**	V14-3 D1-1 J1	V4-72 J5
**SP70**	V5-12 D2-1 J4	V10-96 J1

Dual

Clone	*Igh*	*Igk*
**DP13**	V1-19 D2-3 J4	V10-96 J1
DP11	V1-26 D1-1 J2	V8-19 J5
DP47	V14-1 D2-2 J2	V5-43 J2
**DP69**	V1-53 D6-3 J3	V4-53 J1
**DP76**	V1-64 D1-1 J2	V6-17 J1
**DP105**	V1-64 D2-2 J1	V4-79 J5
**DP156**	V1-64 D2-2 J1	V4-79 J5
DP98	V1-72 D2-3 J2	V8-30 J4
**DP98**	V1-75 D2-1 J2	V4-53 J5
**DP131**	V1-81 D1-1 J2	V5-48 J1

LAH

Clone	*Igh*	*Igk*
**DP79**	V1-15 D2-3 J3	V2-109 J2
**DP25**	V14-2 D4-1 J2	V8-21 J1
**DP7**	V1-50 D1-1 J2	V8-21 J1
**DP60**	V1-50 D1-1 J2	V8-21 J1
**DP70**	V1-50 D2-5 J3	V4-52 J2
**DP104**	V1-50 D2-14 J1	V10-94 J5
**DP119**	V1-50 D2-14 J1	V10-94 J5
**DP75**	V1-69 D1-1 J1	V10-94 J5
**DP111**	V1-69 D1-1 J1	V10-94 J5
**DP114**	V1-69 D1-1 J1	V10-94 J5
**DP129**	V1-69 D1-1 J1	V10-94 J5
**DP146**	V1-69 D1-1 J1	V10-94 J5
**DP157**	V1-69 D1-1 J1	V10-94 J5
**DP144**	V1-69 D2-5 J3	V2-109 J2
**DP149**	V1-69 D2-5 J3	V2-109 J2
**DP55**	V8-12 D1-1 J2	V4-72 J5

Stalk

Clone	*Igh*	*Igk*
**DP14**	V1-76 D2-13 J2	V6-23 J5
**DP44**	V1-76 D2-13 J2	V6-23 J5
**DP41**	V1-81 D2-3 J2	V12-44 J1
**DP134**	V7-3 D2-3 J1	V6-23 J5

Supplementary Fig. 3 Repertoire usage in single or dual-HA binding B cell clones
The *Igh* and *Igk* repertoires in single or dual-HA binding B cell clones isolated from MLN of A/Narita/1/2009 infected mice are indicated. The blue box indicates the LAH binding clones and the red box indicates the rHA2 binding clones. BCR sequences of HA-specific IgG^{+} B cells were obtained by MiSeq and analyzed with the ImMunoGeneTics (IMGT) HighV-QUEST.
.

Supplementary Fig. 4

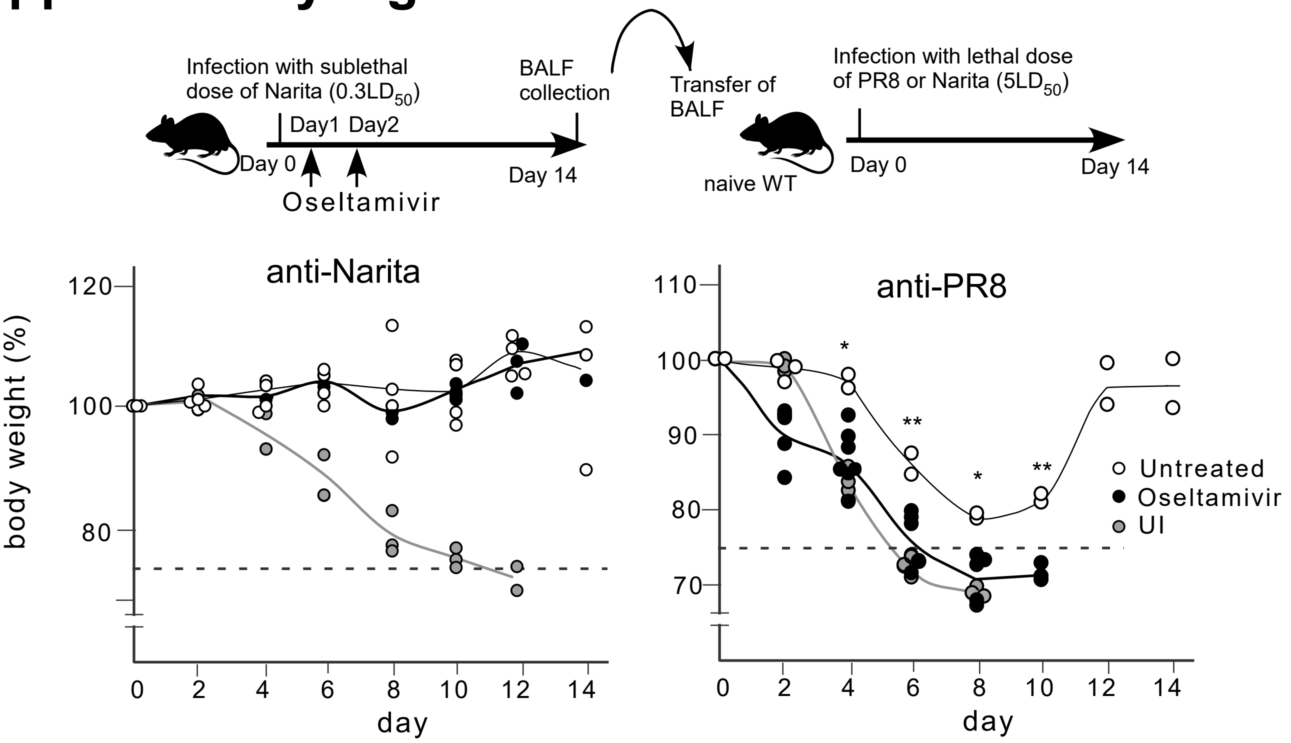

Supplementary Fig. 4 Lack of antibody responses against a heterotypic virus in oseltamivir treated mice

The top panel indicates the design of the BALF transfer experiment for protective antibodies in oseltamivir treated mice. Mice were intranasally infected with A/Narita/1/2009 (0.3LD_{50}) following three days of treatment with 1 mg/head/day of oseltamivir (i.p.). Mice intranasally treated with BALF from mice treated with (black dots) or without (white dots) oseltamivir were challenged intranasally with a lethal dose of A/Narita/1/2009 (left, untreated n=5, treated n=3) or A/PR/8/1934 (right, untreated n=2, treated n=5). The body weight change of mice was monitored until 14 days post-infection. Missing symbols mean that the mice were euthanized, as described in Fig.1. UI represents unimunized. *p<0.05, **p<0.01 by unpaired, two-tailed t-test (Untreated group with Oseltamivir group).

Supplementary Fig. 5

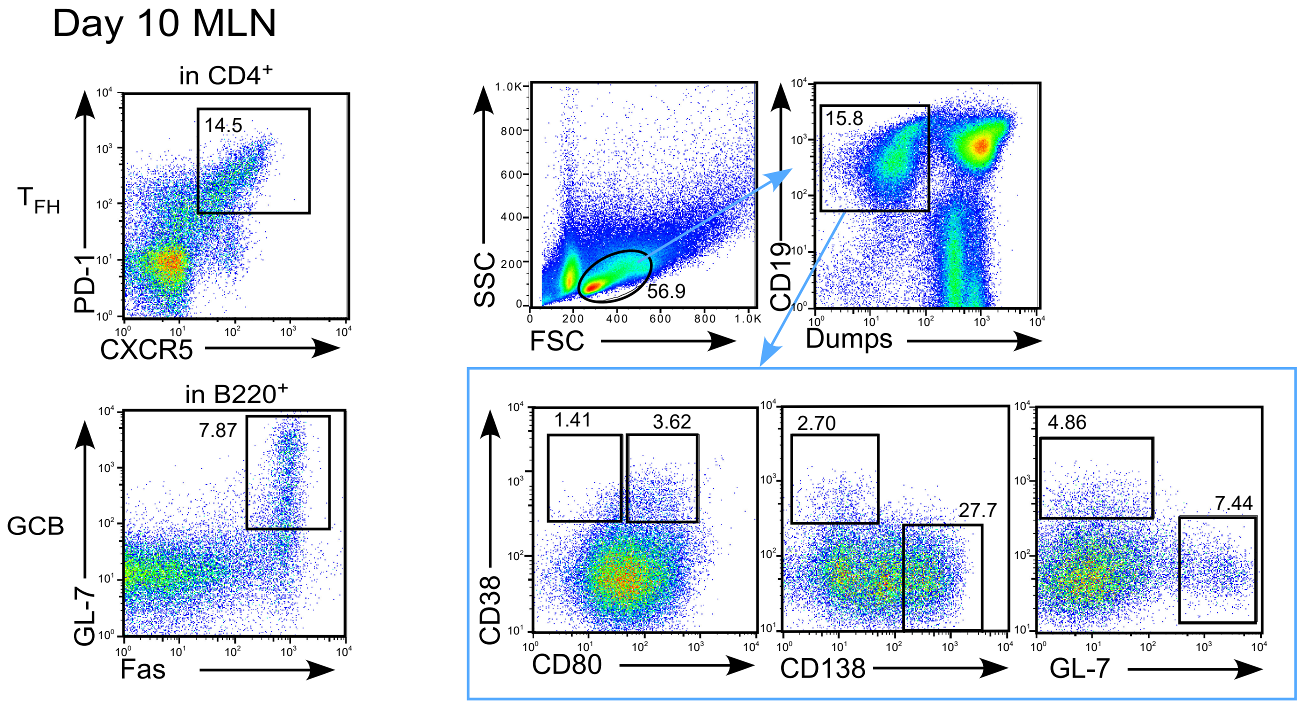

Supplementary Fig. 5 Memory type HA-binding B cells in the MLN from A/Narita/1/2009 infected mice at 10 days post-infection

The percentage of T_{FH} cells, GC B cells, and memory B cells among $B220^{+}IgM^{-}IgD^{-}Dump^{-}$ B cells were analyzed in MLN of mice infected with A/Narita/1/2009 at 10 days post-infection by flow cytometry.

Supplementary Fig. 6

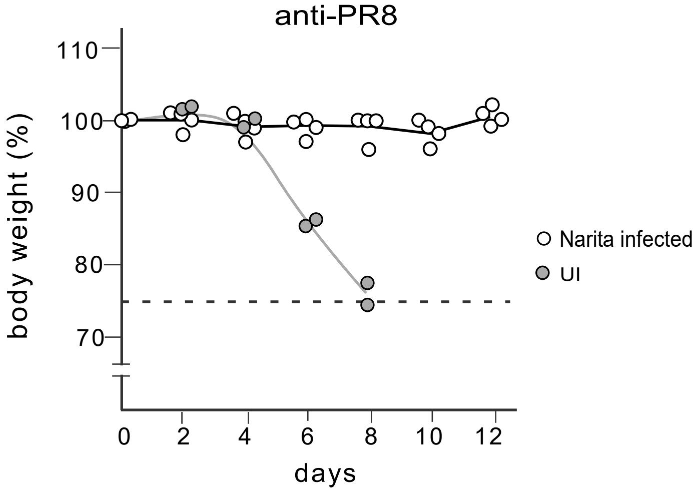

Supplementary Fig. 6 Memory immune response against heterologous virus infection
Mice immunized by intranasal infection with A/Narita/1/2009 were intranasally challenged with a lethal dose of A/PR/8/1934 (n=4) at 40 days post-immunization. The body weight changes were analyzed in the mice with or without (UI n=2) immunization. Missing symbols mean that the mice were euthanized, as described in Fig.1.

Supplementary Fig. 7

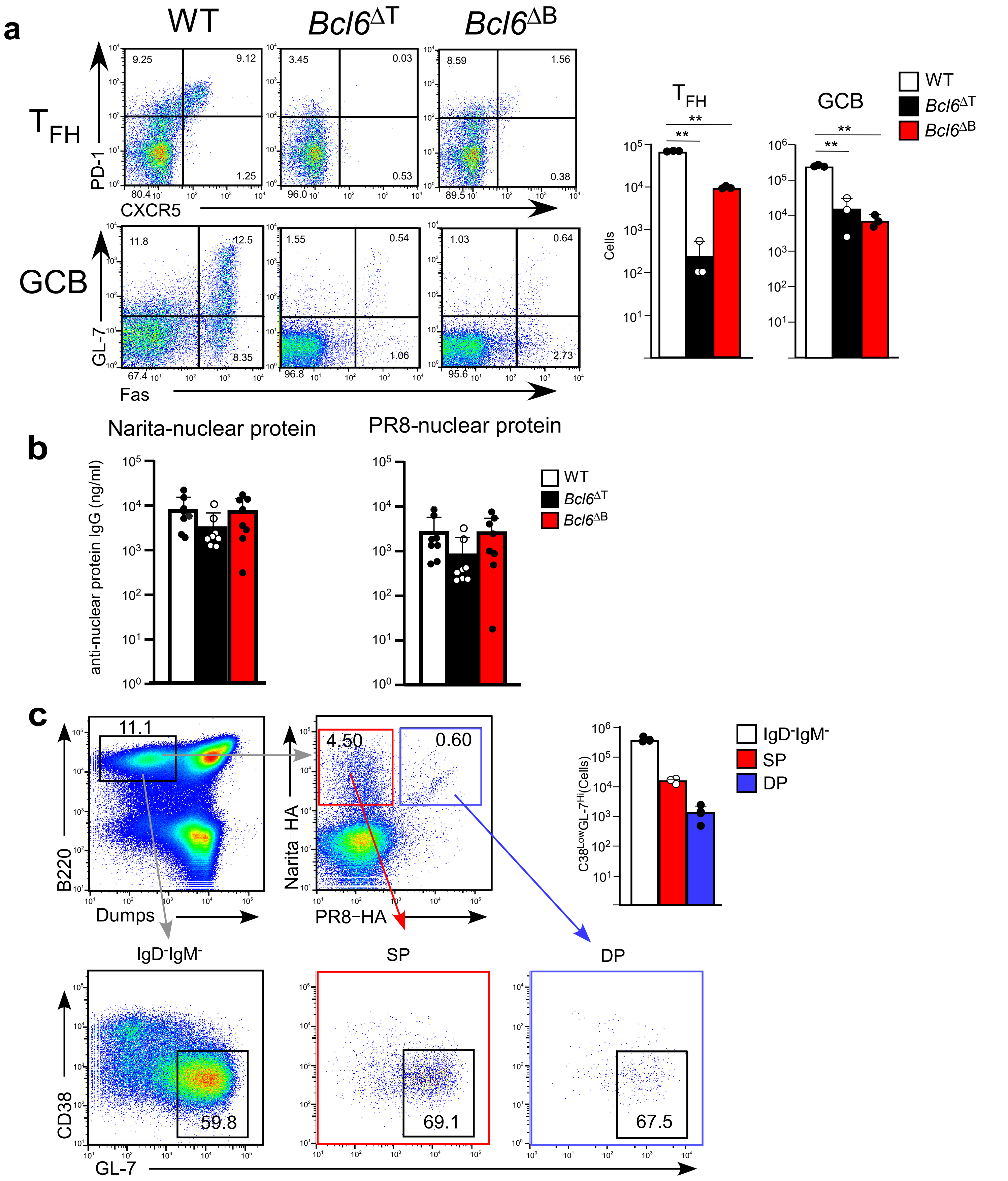

Supplementary Fig. 7 Effect of *Bcl6* deficiency on T_{FH} cells, GC B cells, and viral anti-nuclear protein-specific IgG responses

a, Flow cytometry analysis and percentage of T_{FH} cells and GC B cells in MLN from WT, $Bcl6^{\Delta T}$, and $Bcl6^{\Delta B}$ (n=3 for each) mice infected with A/Narita/1/2009 at 14 days post-infection. Cells were gated on $CD4^+$ (top panels) and $B220^+$ (bottom panels) cell populations for this analysis. Bars represent mean with s.d. of WT (white), $Bcl6^{\Delta T}$(Black), and $Bcl6^{\Delta B}$ (red) mice; ***p*<0.01 by unpaired, two-tailed t-test (WT group with other groups respectively). **b**, Control WT (n=8), $Bcl6^{\Delta T}$(n=8), and $Bcl6^{\Delta B}$ (n=8) mice were infected with A/Narita/1/2009, and serum IgG titers of anti-Narita- or PR8-nuclear (N) protein were measured by ELISA. **c**, $B220^+IgM^-IgD^-Dump^-$ B cells were obtained from MLN of mice infected with A/Narita/1/2009. $CD38^{low}GL\text{-}7^+$ GC-B cells were analyzed for binding to Narita-HA or PR8-HA by flow cytometry.

Supplementary Fig. 8

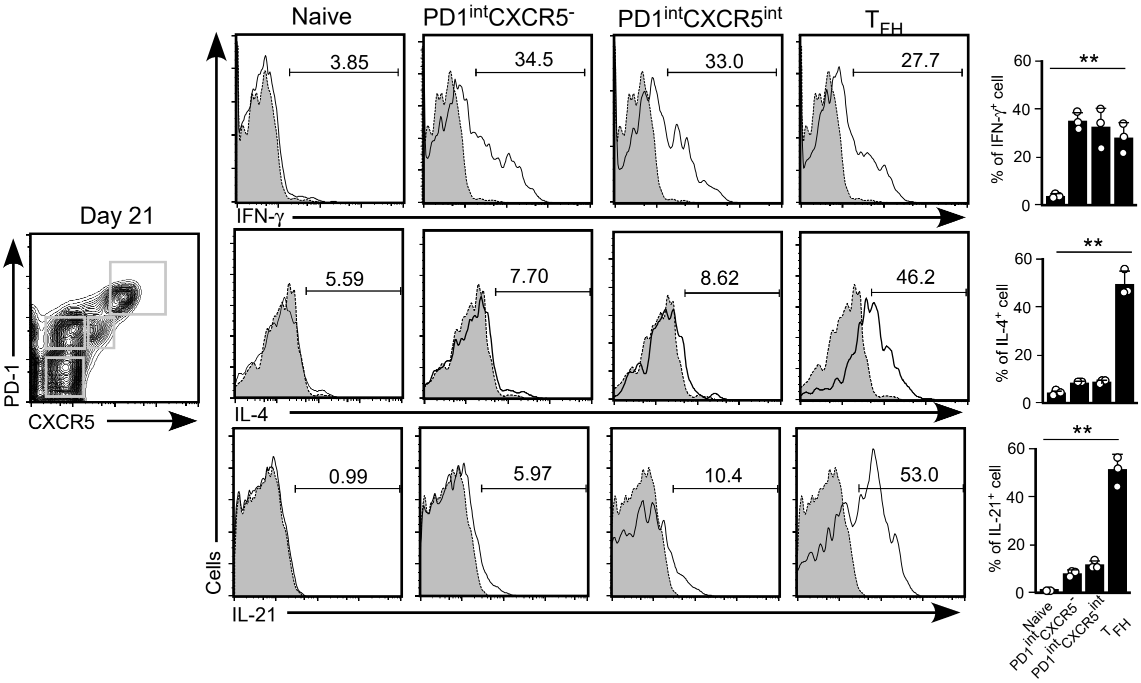

Supplementary Fig. 8 Cytokine production by T_{FH} cells isolated from MLN of A/Narita/1/2009 infected mice

Percentage and Number of IFN-γ-, IL-4- or IL-21-producing T_{FH} cells ($CD4^{+}PD\text{-}1^{+}$ $CXCR5^{+}$) were analyzed in MLN from the IFN-γ-, IL-4- or IL-21 reporter mice (n=3 each) infected with A/Narita/1/2009 at 14-day post-infection. The percentages of IFN-γ-, IL-4- or IL-21-producing T_{FH} cells were determined by flow cytometry analysis. Cells were gated on the $CD4^{+}$ cell population for these analyses. Bars represent mean with s.d.; ***p*<0.01 by unpaired, two-tailed *t*-test (Naive group with T_{FH} group).

Supplementary Fig. 9

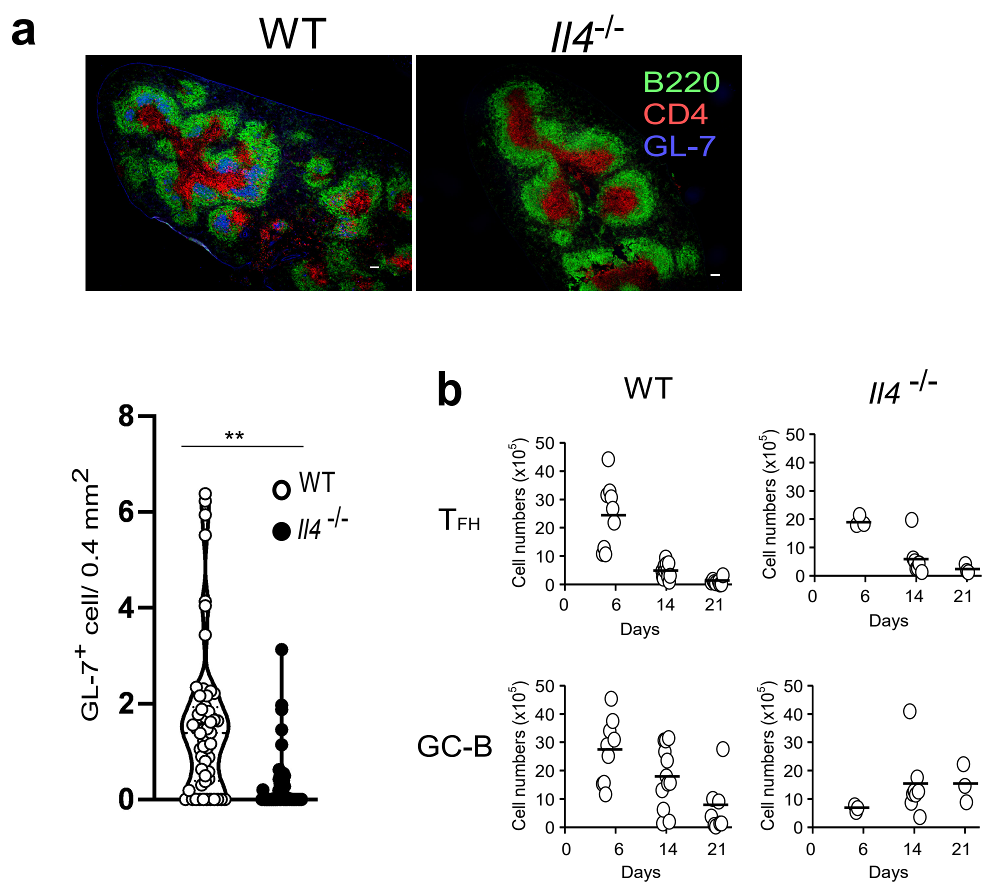

Supplementary Fig. 9 The role of IL-4 signaling in T_{FH} and GC B development

a, WT or *Il4*$^{-/-}$ naive OT-II CD4^{+} T cells were transferred into *Cd28*$^{-/-}$ mice followed by the immunization with OVA. Frozen sections were prepared at 7 days post-immunization and stained for B220, GL-7, and CD4 (top). Each dot in the graph represents the number of the GL-7^{+} B cells localized in a 0.04 mm^{2} square area of a B cell follicle (bottom 46 square areas each). Scale bar indicates 100μm. **b**, The data indicate the number of T_{FH} and GC B cells in the spleen from the *Cd28*$^{-/-}$ mice transferred with naive CD4^{+} T cells from WT or *Il4*$^{-/-}$ mice. The analysis was carried out at days 6 (WT n=8, *Il4*$^{-/-}$ n=3), 14 (WT n=12, *Il4*$^{-/-}$ n=8), 21 (WT n=8, *Il4*$^{-/-}$ n=3) post-immunization with OVA.***p*<0.01 by unpaired, two-tailed *t*-test (WT group with *Il4*$^{-/-}$ group).

Supplementary Fig. 10

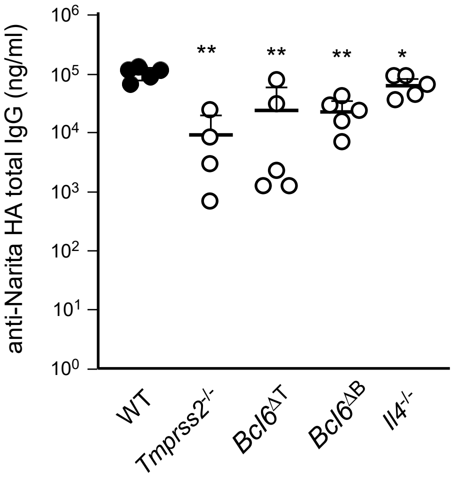

Supplementary Fig. 10 Total anti- A/Narita/1/2009 HA IgG titers in *Tmprss2*, *Bcl6*, and *Il4* deficient mice

Control WT (n=5), *Tmprss2*$^{-/-}$ (n=4), *Bcl6*$^{\Delta T}$ (n=5), *Bcl6*$^{\Delta B}$ (n=5), and *Il4*$^{-/-}$ (n=5) mice were infected with A/Narita/1/2009 and serum IgG titers for anti-Narita-HA were measured by ELISA. *p<0.05, **p<0.01 by unpaired, two-tailed t-test (WT group with other groups respectively).

Supplementary Fig. 11

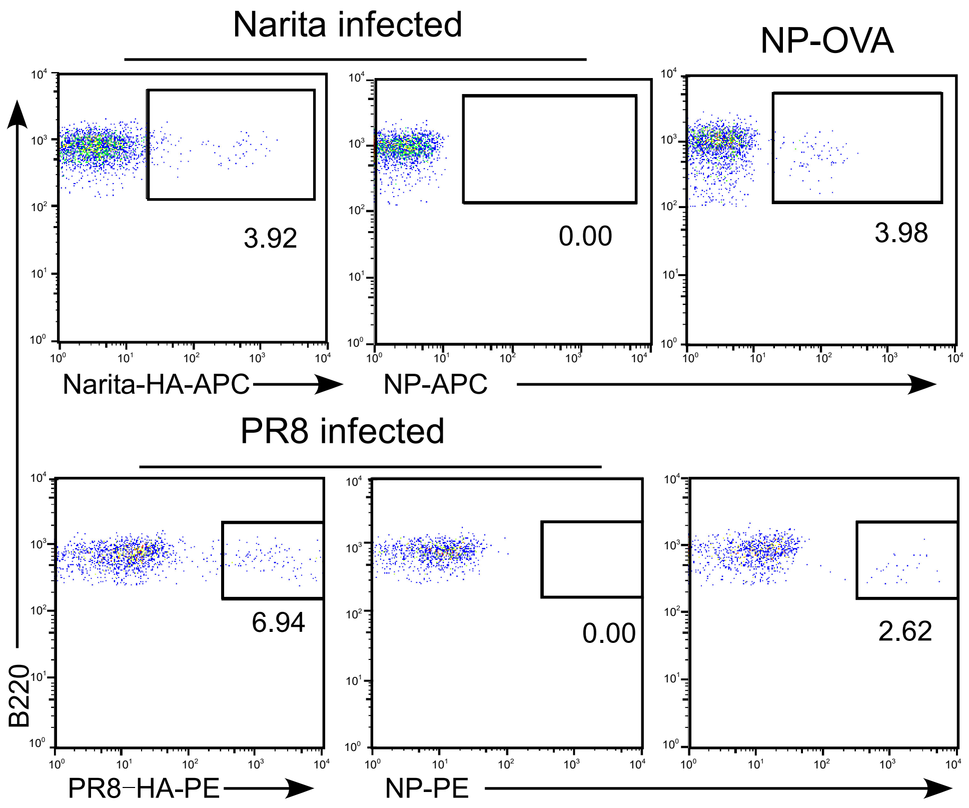

Supplementary Fig. 11 Verification for the specificity of the HA probes
B220^{+}IgM^{-}IgD^{-}Dump^{-} B cells were obtained from mice infected with A/Narita/1/2009 or A/PR/8/1934-HA and from mice immunized with NP$_{16}$-OVA+alum, respectively. Binding to Narita-HA, PR8-HA, or NP$_{29}$-BSA was determined by flow cytometry.

Supplementary Fig. 12

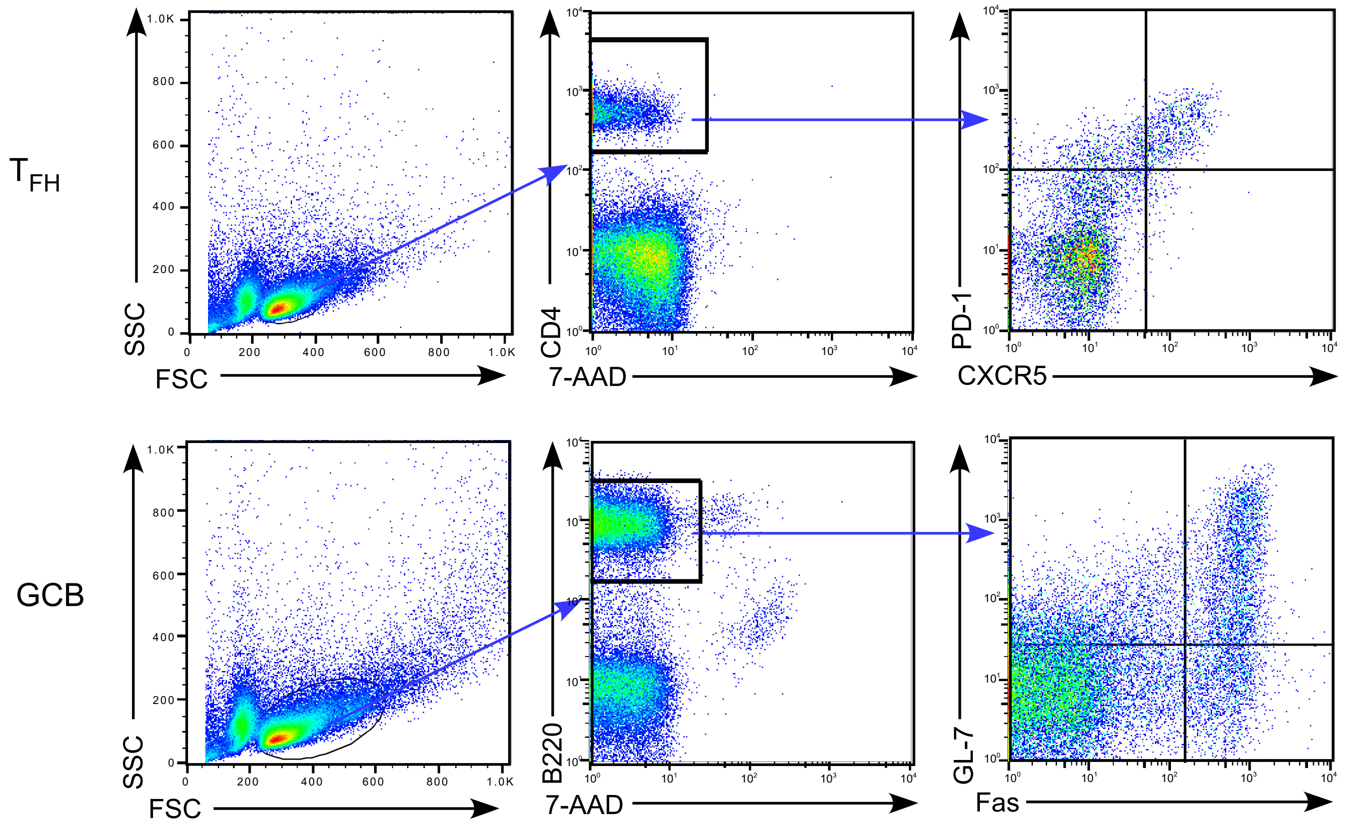

Supplementary Fig. 12 The gating strategy for flow data in Figure 6d, and Supplementary Figure 5, 7a, and 8.

Supplementary Fig. 13

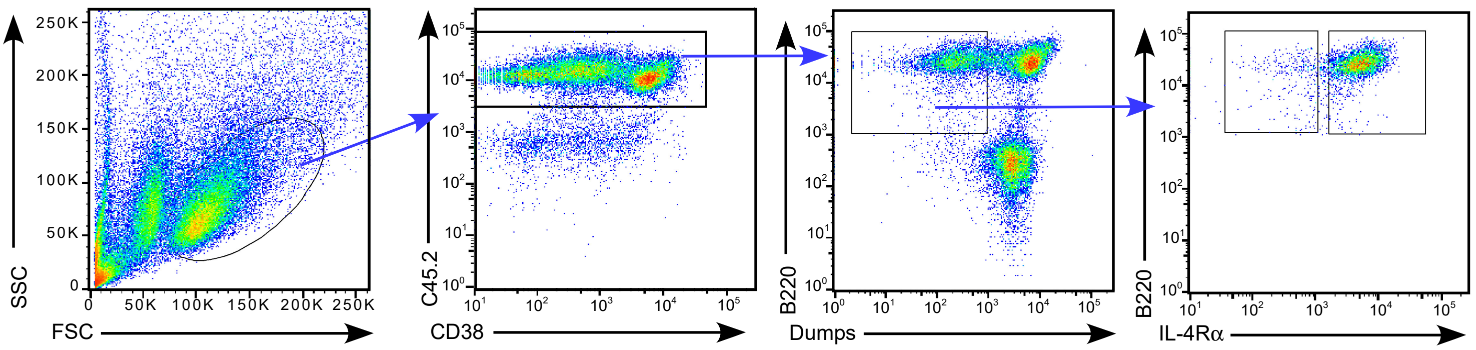

Supplementary Fig. 13 The gating strategy for flow data in Figure 7a.

Supplementary Table 1

Primer use	Primer name	Sequence (5'-3')
KO mice generated by CRISPR-Cas9		
	gRNA-F101	ccaagctttaatacgactcac
	gRNA-R-XbaI	cctctagaaaagcaccgactcggtgccagttgataacg gactagccttattttaacttgctatttctagctctaaaac
	gRNA-R101	cctctagaaaagcaccgactc
	Cxcr3	ccaagctttaatacgactcactatagcagccaagccat gtaccttggttttagagctagaaatagcaagt
	Ccr7	ccaagctttaatacgactcactatagccccagagcac catggacccgttttagagctagaaatagcaagt
Real-time PCR		
	Actb Forward	ggatgccacaggattccatac
	Actb Reverse	actattggcaacgagcggttc-3’
	Aicda Forward	cgtggtgaagaggagagatagtg
	Aicda Reverse	cagtctgagatgtagcgtaggaa
	Bcl2 Forward	gtcccgcctcttcacctttcag
	Bcl2 Reverse	gattctggtgtttccccgttgg
	Bcl6 Forward	ccggcacgctagtgatgtt
	Bcl6 Reverse	tgtcttatgggctctaaactgct
	Ccnb2 Forward	agctcccaaggatcgtcctc
	Ccnb2 Reverse	tgtcctcgttatctatgtcctcg
	Ccnd2 Forward	gagtgggaactggtagtgttg
	Ccnd2 Reverse	cgcacagagcgatgaaggt
	c-Myb Forward	gcgttggtctgttattgc
	c-Myb Reverse	ttctgtcctcctcttctgt
	c-Myc Forward	gccagccctgagcccctagt
	c-Myc Reverse	gggctgtgcggaggtttgct
	G6PD Forward	ccggaaactggctgtgcgct
	G6PD Reverse	ccaggtcacccgatgcaccc
	Glut Forward	catccttattgcccaggtgttt
	Glut Reverse	gaagacgacactgagcagcaga
	HK2 Forward	tgatcgcctgcttattcacgg
	HK2 Reverse	aaccgcctagaaatctccaga
	Irf4 Forward	tccgacagtggttgatcgac
	Irf4 Reverse	cctcacgattgtagtcctgctt
Ig Sequencing		
	Universal Long Primer	ctaatacgactcactgtatcaacgcagagt
	Universal Short Primer	ctaatacgactcactatagggc
	mC_G	atctccacacacaggggccagtggataga

Supplementary Table 1 Primers used in this study.
