# Supplementary material for: Influenza virus infection expands the breadth of antibody responses through IL-4 signalling in B cells
Source: Nat Commun. 2021 Jun 18;12:3789. doi: 10.1038/s41467-021-24090-z (PMC8213721; doi:10.1038/s41467-021-24090-z)
Supplement: Supplementary file 3 — Reporting Summary [file 41467_2021_24090_MOESM3_ESM.pdf]

nature research

Corresponding author(s): Masato Kubo

Last updated by author(s): Apr 29, 2021

Reporting Summary

Nature Research wishes to improve the reproducibility of the work that we publish. This form provides structure for consistency and transparency in reporting. For further information on Nature Research policies, see our Editorial Policies and the Editorial Policy Checklist.

Statistics

For all statistical analyses, confirm that the following items are present in the figure legend, table legend, main text, or Methods section.

n/a	Confirmed	
☐	☒	The exact sample size (n) for each experimental group/condition, given as a discrete number and unit of measurement
☐	☒	A statement on whether measurements were taken from distinct samples or whether the same sample was measured repeatedly
☐	☒	The statistical test(s) used AND whether they are one- or two-sided<br>*Only common tests should be described solely by name; describe more complex techniques in the Methods section.*
☐	☒	A description of all covariates tested
☐	☒	A description of any assumptions or corrections, such as tests of normality and adjustment for multiple comparisons
☐	☒	A full description of the statistical parameters including central tendency (e.g. means) or other basic estimates (e.g. regression coefficient) AND variation (e.g. standard deviation) or associated estimates of uncertainty (e.g. confidence intervals)
☐	☒	For null hypothesis testing, the test statistic (e.g. F, t, r) with confidence intervals, effect sizes, degrees of freedom and P value noted<br>*Give P values as exact values whenever suitable.*
☒	☐	For Bayesian analysis, information on the choice of priors and Markov chain Monte Carlo settings
☒	☐	For hierarchical and complex designs, identification of the appropriate level for tests and full reporting of outcomes
☒	☐	Estimates of effect sizes (e.g. Cohen's d, Pearson's r), indicating how they were calculated

Our web collection on statistics for biologists contains articles on many of the points above.

Software and code

Policy information about availability of computer code

Data collection	We did not use any software for data collection.
Data analysis	Flowcyto data were analyzed with Flowjo software (BD Biosciences). BCR sequencing data were analyzed with the ImMunoGeneTics (IMGT) HighV-QUEST (http://www.imgt.org/). BCR sequenced reads were analyzed with the IMGT HighV-QUEST (http://www.imgt.org/) and tools available on the Galaxy platform (https://usegalaxy.org). Statistical comparisons between groups were performed using Prism version 8.0.2 (Graph Pad Software, San Diego, CA).

For manuscripts utilizing custom algorithms or software that are central to the research but not yet described in published literature, software must be made available to editors and reviewers. We strongly encourage code deposition in a community repository (e.g. GitHub). See the Nature Research guidelines for submitting code & software for further information.

Data

Policy information about availability of data

All manuscripts must include a data availability statement. This statement should provide the following information, where applicable:

- Accession codes, unique identifiers, or web links for publicly available datasets
- A list of figures that have associated raw data
- A description of any restrictions on data availability

Raw source data are present in the Source Data file in this paper. The sequencing data of BCR in Fig. 3 have been deposited in GEO under the accession number GSE168976.

Field-specific reporting

Please select the one below that is the best fit for your research. If you are not sure, read the appropriate sections before making your selection.

- [x] Life sciences
- [ ] Behavioural & social sciences
- [ ] Ecological, evolutionary & environmental sciences

For a reference copy of the document with all sections, see nature.com/documents/nr-reporting-summary-flat.pdf

Life sciences study design

All studies must disclose on these points even when the disclosure is negative.

Sample size	The sample size used in a study was determined based on the need for it to offer sufficient statistical power.
Data exclusions	We did not any data exclusions.
Replication	We replicate each experiments more than two times and obtained similar results.
Randomization	We randomly picked mice to be used in experiments.
Blinding	We have separated who select the mice for the experiments from who perform the experiments.

Reporting for specific materials, systems and methods

We require information from authors about some types of materials, experimental systems and methods used in many studies. Here, indicate whether each material, system or method listed is relevant to your study. If you are not sure if a list item applies to your research, read the appropriate section before selecting a response.

Materials & experimental systems

n/a	Involved in the study	
☐	☒	Antibodies
☒	☐	Eukaryotic cell lines
☒	☐	Palaeontology and archaeology
☐	☒	Animals and other organisms
☒	☐	Human research participants
☒	☐	Clinical data
☒	☐	Dual use research of concern

Methods

n/a	Involved in the study	
☒	☐	ChIP-seq
☐	☒	Flow cytometry
☒	☐	MRI-based neuroimaging

Antibodies

Antibodies used	anti-influenza virus HA (California strain) Abs (clone: RM01; 1:10,000 dilution, SinoBiological, Beijing, China, 86001-RM01), anti-influenza virus HA (PR8 strain) Abs (clone: R107; 1:10,000 dilution, SinoBiological, 11684-R107), anti-mouse IgG1 Abs (1:10,000 dilution, Southern Biotech, Birmingham, AL, 1070-01 and 1:10,000 dilution, Bethyl, Montgomery, TX, A90-105P), anti-mouse IgG2b Abs (1:10,000 dilution, Bethyl, A90-109P), anti-mouse IgG2c Abs (1:10,000 dilution, AVIVA System Bio, San Diego, CA, OASB01582), anti-mouse IgA Abs (1:10,000 dilution, Bethyl, A90-103A and A90-103P). anti-mouse IgG-AP antibody (1:5000 dilution, Proteintech, Rosemont, IL, SA00002-1), anti-B220 mAbs (clone: RA3-6B2; 1:100 dilution, Biolegend, 103208), anti-CD4 mAbs (clone: RM4-5; 1:100 dilution, Biolegend, 100530), anti-GL-7 mAbs (1:200 dilution, BD Biosciences, 553666), anti-pS6 mAbs (clone: N7-548; 1:200 dilution, BD Biosciences, 560434) mAbs, anti-CD3 mAbs (clone: 145-2C11; 1:200 dilution, Biolegend, 100304), anti-CD4 (clone: GK1.5; 1:1000 dilution, Biolegend, 100423), anti-CD5 (clone: 53-7.3; 1:200 dilution, Biolegend, 100604), anti-CD8a mAbs (clone: 53-6.7; 1:200 dilution, Biolegend, 100704), anti-CD11b mAbs (clone: M1/70; 1:200 dilution, Biolegend, 101204), anti-CD11c mAbs (clone: N418; 1:200 dilution, Biolegend, 117304), anti-CD19 mAbs (clone: 6D5; 1:1000 dilution, Biolegend, 115528), anti-CD38 mAbs (clone: 90; 1:200 dilution, Biolegend, 102714), anti-CD80 mAbs (clone: 16-10A1; 1:200 dilution, Biolegend, 104707), anti-CD45.1 mAbs (clone: A20; 1:250 dilution, Biolegend,110730), anti-CD45.2 mAbs (clone: 104; 1:200 dilution, eBioscience, 11-0454-85), anti-CD49b mAbs (clone: DX5; 1:200 dilution, Biolegend, 108904), anti-Fas/CD95 mAbs (clone: Jo2; 1:500 dilution, BD Biosciences, 554258), anti-CD138 mAbs (clone: 281-2; 1:200 dilution, BD Biosciences, 558626), anti-CXCR4 mAbs (clone: L276F12; 1:400 dilution, Biolegend, 146511), anti-CXCR5 mAbs (clone: L138D7; 1:50 dilution, Biolegend, 145506), anti-F4/80 mAbs (clone: BM8; 1:200 dilution, Biolegend, 123106), anti-GL-7 mAbs (1:200 dilution, Biolegend,144610), anti-Gr-1 mAbs (clone: RB6-8C5; 1:200 dilution, BD Biosciences, 553124), anti-hCD2 mAbs (clone: S5.2; 1:20 dilution, BD Biosciences, 744873), anti-IgD mAbs (clone: 11-26c; 1:200 dilution, Biolegend, 405710), anti-IgM mAbs (clone: RMM-1; 1:200 dilution, Biolegend, 406512), anti-IL-4Ra mAbs (clone: mIL4R-M1; 1:20 dilution, BD Biosciences, 552509), anti-NK1.1 mAbs (clone: PK136; 1:200 dilution, Biolegend, 108704), anti-PD-1 mAbs (clone: RMP1-30; 1:200 dilution, Biolegend, 109110), anti-TER-119 mAbs (1:200 dilution, Biolegend, 116204). anti-Ki67 mAbs (clone: 16A8; 1:200 dilution, Biolegend, 652410).
Validation	Specificity of following antibodies was validated with isotype control antibody, anti-B220 mAbs (clone: RA3-6B2), anti-CD4 mAbs (clone: RM4-5; 1), anti-GL-7 mAbs (BD, 553666), anti-pS6 mAbs (clone: N7-548) mAbs, anti-CD3 mAbs (clone: 145-2C11), anti-CD4

(clone: GK1.5), anti-CD5 (clone: 53-7.3), anti-CD8a mAbs (clone: 53-6.7), anti-CD11b mAbs (clone: M1/70), anti-CD11c mAbs (clone: N418), anti-CD19 mAbs (clone: 6D5), anti-CD38 mAbs (clone: 90), anti-CD80 mAbs (clone: 16-10A1), anti-CD45.1 mAbs (clone: A20), anti-CD45.2 mAbs (clone: 104), anti-CD49b mAbs (clone: DX5), anti-Fas/CD95 mAbs (clone: Jo2), anti-CD138 mAbs (clone: 281-2), anti-CXCR4 mAbs (clone: L276F12), anti-CXCR5 mAbs (clone: L138D7), anti-F4/80 mAbs (clone: BM8), anti-GL-7 mAbs (Biolegend,144610), anti-Gr-1 mAbs (clone: RB6-8C5), anti-hCD2 mAbs (clone: S5.2), anti-IgD mAbs (clone: 11-26c), anti-IgM mAbs (clone: RMM-1), anti-IL-4Ra mAbs (clone: mIL4R-M1), anti-NK1.1 mAbs (clone: PK136), anti-PD-1 mAbs (clone: RMP1-3), anti-TER-119 mAbs (Biolegend, 116204). anti-Ki67 mAbs (clone: 16A8). Culture supernatant that did not include target molecule were used for validation following antibodies, anti-influenza virus HA Abs (clone: RM01 and R107), anti-mouse IgG1 Abs (Southern Biotech, 1070-01 and Bethyl, A90-105P), anti-mouse IgG2b Abs (Bethyl, A90-109P), anti-mouse IgG2c Abs (AVIVA, OASB01582), anti-mouse IgA Abs (Bethyl, A90-103A and A90-103P).

Animals and other organisms

Policy information about studies involving animals; ARRIVE guidelines recommended for reporting animal research

Laboratory animals	C57BL/6Jjcl mice were purchased from CLEA Japan (Meguro, Tokyo, Japan). Six-ten weeks old female mice were used for experiments.
Wild animals	We did not use wild animals in this study.
Field-collected samples	We did not use field-collected samples in this study.
Ethics oversight	All mice used in this study were maintained under specific pathogen-free conditions. Room lights are turned on at 7:00 a.m. and turned off at 7:00 p.m. The temperature is 23±2℃ and the humidity is maintained at 50±10%. The animal care was under the guidelines of the RIKEN Yokohama Institute.

Note that full information on the approval of the study protocol must also be provided in the manuscript.

Flow Cytometry

Plots

Confirm that:

☒ The axis labels state the marker and fluorochrome used (e.g. CD4-FITC).

☒ The axis scales are clearly visible. Include numbers along axes only for bottom left plot of group (a 'group' is an analysis of identical markers).

☒ All plots are contour plots with outliers or pseudocolor plots.

☒ A numerical value for number of cells or percentage (with statistics) is provided.

Methodology

Sample preparation	Isolated cells were treated with 2.4G2 antibody in MACS buffer, and then were stained with antibodies in MACS buffer for 30 min at room temperature.
Instrument	FACS calibur and FACS Aria
Software	Flowjo software (BD Biosciences),
Cell population abundance	Cell population abundance is shown as % of parent population.
Gating strategy	Gating strategy is shown in Fig.3a (Lymphocyte population → B220 positive and Dumps (CD4, CD5, CD8a, Gr-1, CD11c, Siglec-F, CD43, NK1.1, F4/80, Ter-119, IgD, IgM, 7AAD) negative), Supplementary Fig 12 (Lymphocyte population → CD4 or B220 positive and 7AAD negative), and Supplementary Fig 13 (Lymphocyte population → CD45.2 positive → B220 positive and Dumps (CD4, CD5, CD8a, Gr-1, CD11c, Siglec-F, CD43, NK1.1, F4/80, Ter-119, IgD, IgM, 7AAD) negative).

☒ Tick this box to confirm that a figure exemplifying the gating strategy is provided in the Supplementary Information.
